# Field evaluation of diagnostic performance of malaria rapid diagnostic tests in western Kenya

**DOI:** 10.1186/s12936-016-1508-y

**Published:** 2016-09-07

**Authors:** Elizabeth W. Wanja, Nickline Kuya, Collins Moranga, Mark Hickman, Jacob D. Johnson, Carolyne Moseti, Lalaine Anova, Bernhards Ogutu, Colin Ohrt

**Affiliations:** 1 Malaria Diagnostics Centre, Kenya Medical Research Institute/United States Army Medical Research Unit, Kenya, Box 54, Kisumu, 40100 Kenya; 2 Division of Experimental Therapeutics, Walter Reed Army Institute of Research, Silver Spring, MD 20910 USA; 3 Madigan Army Medical Center, 9040 Jackson Ave, Tacoma, WA 98431 USA; 4 Translational Medicine International, LLC, 35 Trung Van Road, Hanoi, Vietnam

**Keywords:** Malaria, Microscopy, Rapid diagnostic tests, Performance, Western Kenya

## Abstract

**Background:**

Malaria continues to be a major burden in the endemic regions of Kenya. Health outcomes associated with case management are dependent on the use of appropriate diagnostic methods. Rapid diagnostic tests (RDTs) have provided an important tool to help implement the WHO recommended parasite-based diagnosis in regions where expert microscopy is not available. One of the questions that must be answered when implementing RDTs is whether these tests are useful in a specific endemic region, as well as the most appropriate RDT to use. Data on the sensitivity and specificity of RDT test kits is important information to help guide test selection by national malaria control programmes.

**Methods:**

This study evaluated the diagnostic performance of RDTs including First Response (FR), CareStart (CS), SD Bioline (SD), and Binax Now (BN). The performance of these malaria kits was compared to microscopy, the gold standard, for the detection of malaria parasites. The malaria RDTs were also compared to PCR which is a more sensitive reference test. Five-hundred participants were included in the study through community screening (50 %) and testing suspected malaria cases referred from health facilities.

**Results:**

Of the 500 participants recruited, 33 % were malaria positive by microscopy while 51.2 % were positive by PCR. Compared to microscopy, the sensitivity of eight RDTs to detect malaria parasites was 90.3–94.8 %, the specificity was 73.3–79.3 %, the positive predictive value was 62.2–68.8 %, and the negative predictive value was 94.3–96.8 %. Compared to PCR, the sensitivity of the RDTs to detect malaria parasites was 71.1–75.4 %, the specificity was 80.3–84.4 %, the positive predictive value was 80.3–83.3 %, and the negative predictive value was 73.7–76.1 %. The RDTs had a moderate measure of agreement with both microscopy (>80.1 %) and PCR (>77.6 %) with a κ > 0.6.

**Conclusion:**

The performance of the evaluated RDTs using field samples was moderate; hence they can significantly improve the quality of malaria case management in endemic regions in Kenya by ensuring appropriate treatment of malaria positive individuals and avoiding indiscriminate use of anti-malarial drugs for parasite negative patients.

## Background

Malaria continues to be a life-threatening illness in Kenya [1]. Early and reliable parasite-based diagnosis, treatment and other malaria control programs such as use of insecticide treated bed-nets and indoor residual spray are key to reducing malaria incidence in Kenya, which occurs principally in the Lake Victoria region. An additional driver for accurate laboratory diagnosis is the increasing incidence of *Plasmodium falciparum* resistance to common anti-malarial drugs, which necessitates the use of more expensive and potentially cost-prohibitive combination therapy [2]. The World Health Organization (WHO) emphasizes diagnosis of malaria prior to treatment to avoid indiscriminate use of anti-malarials. However, presumptive treatment of all fevers, such as malaria, is still popular in resource-poor settings due to lack of effective laboratory infrastructure and technical expertise. In these resource-limited settings, empirical treatment often results in overuse of anti-malarial drugs and delays in the diagnosis of other life-threatening febrile illness [3, 4].

The commonly accepted diagnostic method for detecting malaria is microscopic examination of Giemsa-stained blood films. In expert hands, microscopy is highly sensitive (lower limit of parasites down to ~0.0001 % parasitaemia), and very specific. Microscopy can determine the species and stage of circulating parasites, along with parasitaemia to provide data for disease prognosis and monitoring response to treatment [5]. Microscopic examination of thin and thick smears is highly dependent on the microscopist’s skills and requires blinded confirmatory readings to ensure the findings are confirmed. In local laboratories and clinical facilities with or without expert microscopists, the reported results are often variable. In many African countries, malaria expert microscopists are scarce, and microscopy data are often of poor quality. The poor microscopy results are due to a host of factors such as slide preparation techniques, the quality of essential laboratory supplies, condition of the microscope, workload, lack of training, and skills maintenance [5]. Polymerase chain reaction (PCR) methods can be relied upon in the detection of submicroscopic and microscopic parasites with high sensitivity and specificity. A highly sensitive qRT-PCR has a limit of detection of 0.002 P/µL while microscopy has limit of detection of 100 P/µL [6]. Most qPCR methods target different genes in the parasite genome with most detecting 18S-rRNA, although there are many other non-18S-rRNA-qPCR methods being developed. Despite the high performance of PCR, it has several disadvantages in that it is time consuming and requires training because of the procedures involved, furthermore it’s expensive because of the reagents and complex equipment required to run the assay [7].

The introduction of malaria rapid diagnostic tests (RDTs) has enhanced quick examination of blood samples from suspected malaria patients. However, it is not clear which RDT is the most appropriate in different endemic areas with mono and mixed infections of different parasites. RDTs detect *Plasmodium* parasites by an antigen–antibody reaction. The antigens detected in blood include *Plasmodium* lactate dehydrogenase (pLDH), histidine-rich protein II (HRP-2), and *Plasmodium* aldolase [5]. Those that target HRP-2 detect only *P. falciparum* while those that target pLDH and aldolase antigens can detect non-*falciparum* infections. Regardless of the setting, RDTs require minimal operator training, and yield highly reproducible test interpretations. RDT results are normally rapidly available when the physician is actively managing patients as the test can be completed in less than 20 min. They do not require any instrumentation, electricity, special laboratories, or water and these kits are generally cost-beneficial as they provide accurate diagnosis of disease quickly. Rapid diagnosis of disease avoids the consequences of missed diagnoses of malaria, overuse of anti-malarial, and the possible missed diagnosis of another febrile illness. Differences in sensitivity and specificity among RDTs have been reported due to several possible reasons, including exposure to high temperatures and humidity that cause denaturation of antibodies, HRP-2 gene polymorphisms and deletions, operational difficulties, and human error [8].

National policy guidelines for malaria diagnosis in Kenya clearly articulate the role of RDTs as part of effective malaria case management. The Government has procured RDTs in support of its parasite-based diagnosis policy. These RDTs were procured to be used as the primary method for malaria diagnosis in dispensaries and health centres in Kenya [9]. However, the prevalence of malaria is different in various regions of Kenya, and these regional differences may affect the diagnostic performance of a test. Differences in prevalence are brought about by clinical variability which reflects the differences in patient presentation, including high or low disease incidence. All of these variables can cause differences in sensitivity and specificity of a diagnostic test [10]. There are other variables that can influence the performance of RDTs in different regions, such as population differences, differences in the characteristics and genetic variation of the malaria parasite, and diagnostic practice and skills [11]. The successful use of RDTs in different parts of Kenya depends on selecting the best RDT for that particular region. Minimizing the number of false positives and false negatives is clearly of great importance when selecting the most appropriate RDT. Lack of resources limits countries from performing adequate evaluations of RDTs, which can lead to deploying diagnostic tests in geographic areas where their performance is not assured. Evaluations of RDTs for a specific region will help reduce the financial cost associated with their failure in malaria control programmes. Therefore, in order to assist National Malaria Control Programmes in selection of products for specific regions, four kinds of commercially available RDTs were evaluated in terms of their diagnostic performance against gold standard method, microscopy of Giemsa-stained blood smears.

## Methods

### Study area

**Fig. 1:**
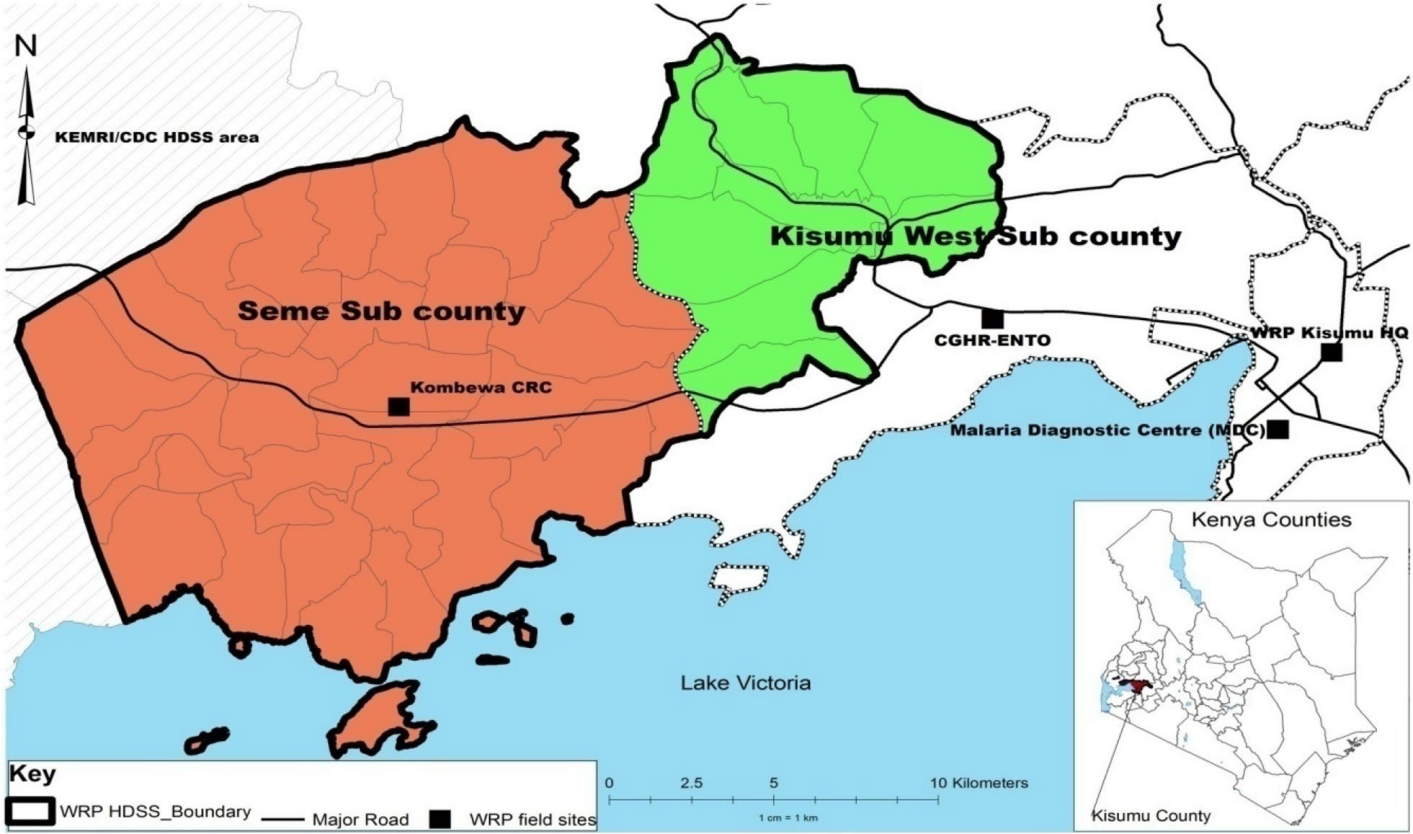
Map of the study area HDSS; also showing the study site (Kombewa CRC)

The study was conducted in the Kombewa Clinical Research Centre (CRC) (Fig. 1), situated 40 km west of Kisumu city, as part of the Kombewa Health and Demographic Surveillance System (HDSS). The site lies between longitudes 34°24′00″E and 34°41′30″E, and latitudes 0°11′30″ N and 0°11′30″S, at an average altitude of 1400 m above sea level. Malaria is holoendemic in this area, and transmission occurs throughout the year. The long rainy season from late March to May produces intense transmission from April to August. The short rainy season from October to December produces another, somewhat less intense, transmission season from November to January [12]. The area has a total of 37 sub-locations and 357 villages. The Kombewa CRC is in close proximity to 24 functioning health facilities, 20 of which are government-owned and four private or faith-based organizations. The HDSS currently monitors a population of 141,956 individuals drawn from 34,718 households [12]. The HDSS population is primarily young, with a mean age of 23 years and 48 % of the population is below the age of 15 years. The age and sex structure has a predominance of young people at the base, and a dearth of older age groups. In 2013, a total of 33,972 cases of illness were reported, a number which represents 24 % of the population. Among these cases, children below the age of 5 years accounted for 27 % of all reported illness. The most prevalent symptoms recorded in the HDSS population were fever (35 %), cold/flu (20 %), cough (18 %), diarrhoea and vomiting (9 %).

### Study design and sampling

The study was conducted between 2013 and 2014 to assess the performance of BinaxNow (Binax Inc, Inverness Medical, ME, USA), SD Bioline (Standard Diagnostics Inc, Korea), Carestart (Access Bio Inc, Monmouth, USA), and First Response (Premier Medical Co Ltd, India). (1) BinaxNow, which targets both *P. falciparum*-specific HRP2 and a pan-malarial antigen, *Plasmodium* aldolase; (2) SD Bioline 05FK50, which targets *P. falciparum*-specific HRP2 only and 05FK60, which targets both *P. falciparum*-specific HRP2 and the pan-malaria antigen pLDH; (3) Carestart IFU G0131, which targets both *P. falciparum*-specific HRP2 and the pan malarial antigen, pLDH, IFU G0141, which targets *P. falciparum*-specific HRP2 only and IFU G0181, which targets both *P. falciparum*-specific HRP2 and pLDH; and, (4) First Response I13FRC, which targets *P. falciparum*-specific HRP2 and First Response I16FRC, which targets both *P. falciparum*-specific HRP2 and the pan-malarial antigen pLDH.

Participants were recruited to the study through the Kombewa HDSS. All patients, irrespective of their age and gender, presenting at the health facilities with a clinical suspicion of malaria were included in the study after taking written informed consent. The study design was in compliance with the STARD methodological guidelines for presentation of diagnostic studies [13].

### Data collection

Data were collected using register forms and laboratory registry notebooks. Intensive training of the study personnel and supervisors was conducted to ensure proper performance of RDT testing and malaria microscopy. Blood specimen from the participants was screened using selected RDTs, and thin and thick blood films were prepared for microscopic examination. All RDTs and blood films for a specific blood specimen were given identical codes for later comparison of the results. Each RDT was performed using the manufacturer’s recommended procedures and results were read between 15 and 20 min while the microscopy films were kept for later examination.

### Rapid diagnostic test interpretation

RDTs contain a nitrocellulose strip with a labeled monoclonal antibody specific for the antigens and are pre-coated with monoclonal antibodies for the test strips and the control line [8]. The interpretation of the results involves the following: for those RDTs detecting HRP2 and pan antigens, three colour bands (bands in the control area, *P. falciparum* area, and pan area) indicated a positive result for *P. falciparum* or mixed infection. For those detecting HRP2 alone, two bands (one band in the control area and another band in the *P. falciparum* area alone) indicated a positive result for *P. falciparum*. When there was only one band in the control area within the result window, this indicated a negative result and the test were deemed invalid if the line in the control area did not appear. Results were recorded using the participants code ID.

### Comparison with Giemsa/microscopy

The microscopic examination of blood films is still a gold standard for the diagnosis of malaria. It has the ability to detect presence or absence of *Plasmodium* parasites by assessing the parasites morphological characteristics and correctly specify the infecting *Plasmodium* species [14]. In this study, thick and thin blood films were prepared, stored in slide boxes, and transported to the Malaria Diagnostic Centre for parasite investigations. The thin and thick blood films were examined at 1000× magnification and results were reported using standard operating procedures [15]. Each blood film was read by two blinded microscopists and if the results showed a discrepancy, a third reading was performed by a third microscopist on the discordant results only. The independent microscopists were blinded in order to assure quality of the microscopic examinations. During slide examination, a slide was regarded as negative after examining 200 high-power fields without finding any parasite. A slide was considered positive if at least one asexual form of parasite was detected in 100 microscopic fields in thick blood film [16]. If a slide was *Plasmodium*-positive, asexual parasites were counted using standard operating procedures of light microscopy as recommended by the WHO. The number of asexual parasites per 200 white blood cells (WBCs), or 500 WBCs for low density infections, were used to compute the number of asexual parasites per μL of blood, assuming a standard count of 8000 WBCs per μL of blood [15]. Performance of RDTs was compared against Giemsa/microcopy results.

### Real-time PCR

The QIAamp DNA Blood Mini Kit (Qiagen, Valencia, CA) was used to extract genomic nucleic acids from whole/frozen blood samples. Amplification and real-time measurements was performed in the Applied Biosystems 7500 analytical PCR system (Life technologies Grand Island, NY, USA). The thermal profile for qPCR was: Reverse Transcription step of 30 min at 50 °C, denaturation step of 10 min at 95 °C and 40 cycles of amplification for 15 s at 95 °C, and annealing for 1 min at 60 °C. For the reaction, 1 µl of template was added to 9 µl of reaction master mix (5 µL 2× Quantitect Mix (QIAgen), 4 mM MgCl_2_, 0.1 µL Reverse Transcription mix 0.4 µM each primer, 0.2 µM probe, and 1.1 µL water). *Plasmodium falciparum* standard curves and positive controls were developed from cultured NF54 parasites while nuclease free water was used as negative control (Life Technologies, Grand Island, NY, USA). The forward primer sequence was 5′-GCTCTTTCTTGATTTCTTGGATG-3′, and the reverse primer sequence was 5′-ATGGCCGTTTTTAGTAAGATCTCGTTCG-3′. The probe sequence was 5′-ATGGCCGTTTTTAGTTCGTG-3′ labeled with reporter dye (5′FAM;-6-carboxyfluorescein) and quencher dye (3′TAMRA:-6-carboxytetramethyl-rhodamine). Primer sets were designed based on highly conserved *Plasmodium* genus sequence (18 s rRNA) so as to amplify all units of rRNA distributed in all chromosomes: 1, 5, 7, 11 and 13. Primer3 v.0.4.0 web based software (http://frodo.wi.mit.edu/primer3/) was used to design the primers.

### Data analysis

Data were recorded on register forms and entered in a Microsoft Excel database (Microsoft Corp, Redmond, Washington, USA) in a secure computer at the Malaria Diagnostics Centre. The sensitivity, specificity and predictive values [calculated with 95 % confidence interval (CI)] of the RDTs compared to Giemsa microscopy and RT-PCR as the reference methods for *Plasmodium* parasite detection. Graph Pad Prism v5.01 (Graph Pad Software, CA, USA) utilizing the two-tailed Fisher’s exact test was used for analysis. Inter-test agreement for both results of positive and negative readings was expressed by the percentage of overall agreement and kappa statistic (κ) for the agreement between malaria RDTs and the reference methods.

## Results

### Characteristics of study participants

Evaluation of BinaxNow, SD Bioline, Carestart, and First Response was based on 500 study participants recruited through the Kombewa HDSS via community screening (~50 %) and suspected malaria cases referred from health facilities. The study participants included children <12 years (58 %, n = 290), and the remainder were ranged in age from 13 to 64 years (42 %, n = 210). The male participants were a minority of participants (36 %, n = 181) while the majority of the participants were female (64 %, n = 319). Adult women who were not pregnant comprised 39 % of the female population (39 %, n = 196), one was pregnant, and the remainder were children. The number of *Plasmodium*-positive cases was 33 % (166/500) by the gold standard Giemsa/microscopy, while 51 % (256/500) were positive by RT-PCR. The eight RDTs detected 44–48 % malaria positive cases and 52–56 % malaria negative cases (Table 1). Of the 166 microscopy positive cases, 84 % (139/166) were *P. falciparum*, and 13 % (22/166) were mixed species of *P. falciparum* and *Plasmodium malariae.*

**Table 1:** Results for Giemsa microscopy, RT-PCR and malaria RDTs for the detection of *Plasmodium* parasites

Method	Positive, N (%)	Negative, N (%)
Giemsa microscopy	166 (33)	334 (67)
RT-PCR	256 (51)	244 (49)
BinaxNOW 665-025	221 (44)	269 (54)
SD Bioline 05FK60	240 (48)	260 (52)
SD Bioline 05FK50	233 (48)	248 (52)
First Response I16FRC	220 (44)	280 (56)
First Response I13FRC	231 (46)	269 (54)
CareStart G0131	232 (46)	268 (54)
CareStart G0181	230 (46)	270 (54)
CareStart G0141	236 (47)	264 (53)

### Performance of eight malaria RDTs as compared to the gold standard Giemsa/microscopy

The sensitivity and specificity of RDTs to detect *P. falciparum* (HRP2-band) against microscopy was relatively similar across all the eight malaria RDTs tested. The sensitivity was shown to be >90 % while the specificity was shown to be >73 % when compared to microscopy. The SD Bioline 05FK50 kit had the highest sensitivity of 94.8 % (89.9–97.7) while First Response I16FRC kit had the lowest sensitivity of 90.4 % (84.8–94.4). The specificity of all the eight malaria kits was >73 % when compared to microscopy. The BinaxNOW 665-025 kit had the highest specificity of 79.3 % (74.6–83.6) while SD Bioline 05FK50 kit had the lowest specificity of 73.3 % (68.1–77.9) (Table 2).

**Table 2:** Performance comparison of malaria RDTs against Giemsa microscopy for the detection of *Plasmodium falciparum*

Malaria RDTs	Sensitivity %, 95 % CI	Specificity %, 95 % CI	Predictive value		Likelihood ratio		Methods agreement %	κ value
			Positive test %, 95 % CI	Negative test %, 95 % CI	Positive test %, 95 % CI	Negative test %, 95 % CI		
BinaxNOW 665-025	*91.6*	*79.3*	*68.8*	*94.9*	*4.4*	*0.1*	*83.4*	*0.7*
	86.3–95.3	74.6–83.6	62.2–74.8	91.7–97.2	3.6–5.5	0.1–0.2		0.6–0.7
SD Bioline 05FK60	*93.4*	*74.6*	*64.6*	*95.8*	*3.7*	*0.1*	*80.8*	*0.6*
	88.5–96.6	69.5–79.1	58.2–70.6	92.6–97.9	3.0–4.4	0.1–0.2		0.5–0.6
SD Bioline 05FK50	*94.8*	*73.3*	*62.2*	*96.8*	*3.5*	*0.1*	*80.1*	*0.6*
	89.9–97.7	68.1–77.9	55.7–68.5	93.8–98.6	2.9–4.3	0.0–0.1		0.5–0.6
First Response I16FRC	*90.3*	*79.0*	*68.2*	*94.3*	*4.3*	*0.1*	*82.8*	*0.6*
	84.8–94.4	74.3–83.3	61.6–74.3	90.9–96.7	3.5–5.3	0.1–0.2		0.5–0.6
First Response I13FRC	*92.8*	*76.9*	*66.7*	*95.5*	*4.0*	*0.1*	*82.2*	*0.6*
	87.7–96.2	72.1–81.4	60.2–72.2	92.3–97.7	3.3–4.9	0.1–0.2		0.5–0.6
CareStart G0131	*93.4*	*76.9*	*66.81*	*95.9*	*4.0*	*0.1*	*82.4*	*0.6*
	88.5–96.6	72.1–81.4	60.4–72.8	92.8–97.9	3.3–4.9	0.1–0.2		0.5–0.6
CareStart G0181	*92.2*	*76.9*	*66.5*	*95.2*	*4*	*0.1*	*82.0*	*0.6*
	86.9–95.8	72.1–81.4	60.0–72.6	91.9–97.4	3.3–4.9	0.1–0.2		0.5–0.6
CareStart G0141	*93.4*	*75.8*	*65.7*	*95.8*	*3.9*	*0.1*	*81.6*	*0.6*
	88.5–96.6	70.8–80.3	59.2–71.7	92.7–97.9	3.2–4.7	0.1–0.2		0.5–0.6

*CI* confidence interval, *RDTs* Rapid diagnostic test

The eight malaria RDTs had a positive predictive value of >62 % but a very high negative predictive value of >94 % when compared to microscopy. The positive predictive value range was between 62.2 % (55.7–68.5) for SD Bioline 05FK50 kit to 68.8 % (62.2–74.8) for BinaxNOW kit. The highest negative predictive value was 96.79 % (92.8–98.6) for the SD Bioline 05FK50 kit. The positive likelihood ration range was 3.5–4.4 while the negative likelihood ratio range was 0.07–0.12 (Table 2).

The RDTs showed a moderate measure of agreement (>80 %) when compared to microscopy, the gold standard for malaria diagnosis (Table 2). The FDA-approved BinaxNOW 665-025 RDT had a higher per cent agreement than the other RDTs: 83.4 % (κ = 0.655). The majority of RDTs used in Kenya are CareStart, and all the three had almost the same measure of agreement; G0181 (82.0 %, κ = 0.63), G0141 (81.6 %, κ = 0.625) and G0131 (82.4 %, κ = 0.639). SD Bioline 05FK50 had 80.08 % (κ = 0.597) and SD Bioline 05FK60 had almost the same 80.8 % (κ = 0.611). First Response I16FRC had 82.8 % (κ = 0.642) while First Response I13FRC 82.2 % (κ = 0.635) (Table 2).

Density dependent sensitivity was observed across all the malaria RDTs. Parasite densities were classified into five groups according to microscopy positive results (n = 166) <200 P/µL (n = 9), 200–500 P/µL (n = 29), 500–2000 P/µL (n = 42), 2000–5000 P/µL (n = 29), >5000 P/µL (n = 38). At <200 P/µL (n = 9), the sensitivity ranged between 72 and 89 % and increased to >93 % at 200–500 P/µL. At very high parasite densities >5000 P/µL, all the RDTs had 100 % sensitivity indicating their ability to correctly detect *P. falciparum* in patient with high levels of the parasite (Table 3).

**Table 3:** Sensitivity of malaria RDTs as compared to Giemsa Microscopy classified by parasite densities

	<200 P/µL (n = 29)	200–500 P/µL (n = 29)	500–2000 P/µL (n = 42)	2000–5000 1P/µL (n = 29)	>5000 P/µL (n = 38)
BinaxNOW 665-025 (%)	79	93	90	96	100
SD Bioline 05FK60 (%)	86	93	93	96	100
SD Bioline 05FK50 (%)	89	96	100	100	100
First Response I16FRC (%)	72	93	90	96	100
First Response I13FRC (%)	83	93	93	96	100
CareStart G0131 (%)	86	93	93	96	100
CareStart G0181 (%)	86	93	88	96	100
CareStart G0141 (%)	83	93	93	100	100

*P/µL* Parasites per microliter

### Performance of eight malaria RDTs as compared to RT-PCR as the reference test

The performance of the RDTs to detect *P. falciparum* when compared to RT-PCR was similar across all the kits but quite different from microscopy. The sensitivity ranged from 71.1 % (65.1–76.6 %) for First Response I16FRC to 75.4 % (69.6–80.5 %) for SD Bioline 05FK60 and CareStart G0181. The specificity of all the kits was >80 % when compared to PCR results. The highest specificity was 84.8 % (79.7–89.1 %) for BinaxNOW while lowest specificity was 80.8 % (75.2–85.6 %) for SD Bioline 05FK50. Unlike when compared to microscopy, the positive predictive values for the eight RDTs was >83.3 % while the negative predictive value was low at >73.6 %. The positive predictive values ranged from 80.3 % (74.6–85.2 %) for SD Bioline 05FK50 to 83.3 % (77.7–87.9 %) for BinaxNOW. The negative predictive values ranged from 73.6 % (68.0–78.6 %) for First Response I16FRC to 76.1 % (70.5–81.2 %) for SD Bioline 05FK60. The positive likelihood ratio ranged between 3.9 and 4.7 while the negative likelihood ratio was at 0.3. The RDTs showed a moderate measure of agreement (>77 %) when compared to RT-PCR as a more sensitive reference method. CareStart had the higher agreement value of 78.8 % while First Response had the lower agreement of 77.6 %. All the RDTs had a relative agreement with PCR at κ > 0.6 (Table 4).

**Table 4:** Performance comparison of malaria RDTs against RT-PCR for the detection of *Plasmodium falciparum*

	Sensitivity %, 95 % CI	Specificity %, 95 % CI	Predictive value		Likelihood ratio		Methods agreement %	κ, 95 % CI
			Positive test %, 95 % CI	Negative test %, 95 % CI	Positive test %, 95 % CI	Negative test %, 95 % CI		
BinaxNOW 665-025	*71.9*	*84.8*	*83.3*	*74.2*	*4.7*	*0.3*	*78.2*	*0.6*
	65.9–77.3	79.7–89.1	77.7–87.9	68.6–79.2	3.5–6.4	0.3–0.4		0.5–0.6
SD Bioline 05FK60	*75.4*	*81.1*	*80.4*	*76.1*	*3.9*	*0.3*	*78.0*	*0.6*
	69.6–80.5	75.6–85.7	74.8–85.2	70.5–81.2	2.9–5.1	0.3–0.4		0.5–0.6
SD Bioline 05FK50	*74.8*	*80.8*	*80.3*	*75.4*	*4.0*	*0.3*	*79.0*	*0.6*
	68.9–80.5	75.2–85.6	74.6–85.2	69.6–80.54	3.1–5.3	0.3–0.4		0.5–0.7
First Response I16FRC	*71.1*	*84.4*	*82.7*	*73.6*	*4.6*	*0.3*	*77.6*	*0.6*
	65.1–76.6	79.3–88.7	77.1–87.5	68.0–78.6	3.4–6.2	0.3–0.4		0.5–0.6
First Response I13FRC	*73.8*	*82.8*	*81.8*	*75.1*	*4.3*	*0.3*	*78.2*	*0.6*
	67.9–79.1	77.5–87.3	76.2–86.6	69.4–80.1	3.2–5.7	0.3–0.4		0.5–0.6
CareStart G0131	*73.8*	*82.4*	*81.5*	*75.0*	*4.2*	*0.3*	*78.0*	*0.6*
	67.9–79.1	77.0–86.9	75.7–86.3	69.4–80.1	3.2–5.5	0.3–0.4		0.5–0.6
CareStart G0181	*72.3*	*81.6*	*80.4*	*73.7*	*3.9*	*0.3*	*76.8*	*0.6*
	66.4–77.7	76.1–86.2	74.7–85.4	68.0–78.9	2.9–5.2	0.3–0.4		0.5–0.6
CareStart G0141	*75.4*	*82.4*	*81.8*	*76.1*	*4.3*	*0.3*	*78.8*	*0.5*
	69.6–80.5	77.0–86.9	76.3–86.5	70.5–81.2	3.2–5.7	0.3–0.4		0.5–0.6

*CI* Confidence interval, *RDTs* Rapid diagnostic test

## Discussion

In this study, the performance of different RDT kits (BinaxNow, First Response, CareStart, and SD Bioline) was evaluated using 500 samples from febrile patients (n = 500). This study reproduces the actual performance these RDTs would be expected to provide under real-world field conditions, complexities and physical stresses. Sensitivities of these kits was shown to be >90 %, specificities were shown to be >73 %, PPV was shown to be >62 %, and NPV was shown to be >94 %, and >80 % agreement with microscopy was shown by these RDT assays. The data from this study are consistent with other published work when RDTs are compared to microscopy. For example, a study in Central Africa Republic showed that SD Bioline antigen (HRP2) had a sensitivity range of 88.1–95.4 %, specificity range of 76.2–87.2 %, PPV of 0.8–89.8, and a NPV of 85.0–94.1 [17]. A study by Maltha et al. [18], in a reference setting showed the sensitivity of the CareStart Malaria HRP-2/pan kit for the detection of *P. falciparum* to be 84.8–92.0 %.

Field and laboratory evaluations in India for the First Response malaria antigen HRP2/pan kit showed a sensitivity ranging from 89.5 to 97.7 % and specificity ranging from 63.6 to 75.6 % for detection of *P. falciparum* [19]. A study conducted in a health centre in Dindori, India, which is a highly malaria-endemic area, examining First Response demonstrated the sensitivity of range from 89.5 to 97.7 % and specificity from 63.6 to 75.6 %, with PPV ranging from 56.5 to 70.3 %, NPV ranging from 91.9 to 98.4 %, and an agreement with microscopy of 78.8 % (κ-0.58) [20].

The sensitivity of RDTs in this study was shown to increase with increase in parasite density. This implies the inability of RDTs to reliably detect malaria parasites at very low parasites densities. Performance of BinaxNow in a large field trial by Gasser et al. showed an overall sensitivity of 95 % for detection of *P. falciparum,* however, stratified *P. falciparum* sensitivity calculations indicated a sensitivity of 93 % (*P. falciparum* >500–1000/µL), 89 % (*P. falciparum* >100–500/µL), and 54 % (*P. falciparum* >0–100/µL) [21]. An evaluation of BinaxNow in Toronto General Hospital, Canada on 256 patients travelling from malaria-endemic countries showed that BinaxNow had a sensitivity of 96 % for infections with a parasite density >100/µL but decreased to 75 % for densities <100/µL when compared to microscopy [22].

The WHO recommends that RDTs should have a sensitivity of >95 % and a specificity of >90 % [8], and the evaluated RDTs demonstrated a relatively strong sensitivity ranging from 90.36 to 94.77 %. Hence, they can be considered as clinically useful diagnostic tools for ruling out malaria. BinaxNow was tested in another study at the bedside and in the clinical laboratory for diagnosis of malaria in 542 patients by Wiese et al. [23] and the sensitivity was shown to be 88 % at the bedside and 95 % in the laboratory. Importantly, the difference in sensitivity between assays at the bedside and in the laboratory may explain why field diagnosis can show a lower sensitivity than recommended by the WHO.

The relatively high sensitivity demonstrated in this study decreased the likelihood of RDTs incorrectly identifying a patient as a false negative, hence showing a substantive ability to identify patients who truly had malaria. A cross-sectional study conducted in Blantyre District in Malawi assessed the performance of HRP2 RDTs (SD Bioline Malaria, First Response, Paracheck, ICT Malaria,) and overall, there were 44/633 (7 %) false-negative RDT results compared to microscopy [24].

The results showed a lower specificity as compared to WHO-recommended specificity of an effective RDT, and this is probably due to prior infection and subsequent effective treatment. The exclusion criteria included malaria treatment within the previous 2 weeks; some participants may have been treated for malaria; however the HRP-2 detected by these RDTs may have persisted in the blood stream before being completely cleared from the blood. A persistent parasite antigenaemia of HRP-2 has been demonstrated; a study on 240 patients with *P. falciparum* mono-infection was done after anti-malarial treatments using ICT malaria and optimal-IT assays; 82.1 % of the patients demonstrated persistent antigenaemia for the HRP2 antigen up to 14 days of follow-up [25]. Others studies have shown that that it can last for up-to 28 days while others suggest that it has a median of 20 days [26, 27]. The second explanation for the high rate of false-positive RDT results may be due to malaria cases with parasite densities below the detection level of microscopy. Examination of the blood slides by microscopy may not detect the parasites due to very low parasitaemia, but the RDTs may test positive because they detect antigens from the parasites [28].

The disease prevalence in this endemic Lake Victoria region is known to be approximately 37 % [9]. Evaluations of the 500 participants indicated a prevalence of 33 %, making the positive and negative predictive values significant. The high NPV shown (>94 %) in this study will lead to RDTs being used with confidence to confirm negative test patients as non-malaria patients. The risk of missing detectable malaria parasites in an infected individual is small for all the RDTs evaluated [20]. Although the test performances of the RDTs were characterized by low PPVs which indicates over-diagnosis this can produce high estimates of malaria morbidity, inflate treatment costs, and create misperceptions of therapeutic failures when fever is due to other illness. This may lead to avoidable, drug-related, adverse events and contribute to unnecessary drug pressure, thereby enhancing selection or drug resistance [3]. There was a small increase (a range of 3.54–4.43) in the likelihood of a positive test result in participants who had malaria compared to participants who did not have malaria. There was a moderate to large decrease (a range of 0.12–0.07) in the likelihood of a negative result in participants who had the disease compared to those without the disease.

The performance of RDTs compared to PCR was quite different than when compared to microscopy. The sensitivity of RDTs was lower at 71.1–75.4 % when compared to PCR but >90 % when compared to microscopy. The specificity of RDTs in our results is almost similar other studies [18, 29–31]. This indicates that the ability of the RDTs to detect malaria positive individuals is heightened when compared to microscopy simply because they have almost a similar detection limit (100 P/µL). A more sensitive method, PCR has the capability of detecting parasites as low as 0.002 P/µL implying that lower sensitivity range observed could be due to submicroscopic infections in the population. Individuals with submicroscopic infections can significantly contribute to plasmodium transmission dynamics by acting as human infectious reservoirs [32, 33]. The levels of infections are known to vary due to several reasons that correlate with malaria transmission intensity. These factors include the mosquito population which accelerate the rates of re-infection, acquired immunity after living in a malaria endemic regions, multiplicity of clonal subtypes of the parasite, and partially successful treatments [32].

The opposite was observed for specificity whereby the levels were higher when compared to PCR (80.8–84.8 %) than microscopy (73.3–79.3 %). The specificity of RDTs in our results is almost similar other studies [18, 29–31]. This indicates that the when compared the PCR; they have a higher ability to actually detect a malaria negative patient. As the malaria prevalence decreases [1], the ability of the diagnostic tools to correctly identify a malaria negative individual will be more relevant. This will also be important in low transmission areas where accurately identifying the presence of malaria parasites ensures accurate treatment with ACT [33] while absence of the disease ensures the correct diagnosis of the underlying disease. The errors associated with RDTs such as false positives, false negatives, inability to detect submicroscopic infections, persistent antigenaemia of HRP2, and polymorphisms of HRP2, will require PCR to complement their usage at the point of care [5, 8, 28]. This is because PCR has the ability to discriminate very low parasite density results, it can detect persistent antigenaemia, and it has very high sensitivity and specificity [6].

## Conclusion

Rapid diagnostic tests can be applied in a number of settings with a great potential for impact on public health where they can significantly improve the quality of malaria case management in areas where expert microscopy or PCR methods are not available. This study demonstrated that CareStart, SD Bioline, BinaxNow, and First Response RDTs performed reasonably well in this endemic region for the detection of *P. falciparum*. More evaluations should be carried out around the country, particularly in non-endemic areas with returning migrant workers from endemic areas as well as in epidemic highland areas. After implementation and deployment of RDTs in health facilities, subsequent research should be done on their impact and cost effectiveness in providing reliable diagnosis at the point of care.
